# Whole-exome sequencing reveals novel mutations and epigenetic regulation in hypopharyngeal carcinoma

**DOI:** 10.18632/oncotarget.19674

**Published:** 2017-07-28

**Authors:** Ping Wu, Honglong Wu, Yaoyun Tang, Shi Luo, Xing Fang, Chubo Xie, Jian He, Suping Zhao, Xiaofeng Wang, Jiajia Xu, Xi Chen, Dongfang Li, Huanming Yang, Jian Wang

**Affiliations:** ^1^ Department of Otorhinolaryngology Head & Neck Surgery, Province Key Laboratory of Otolaryngology Critical Diseases, Xiangya Hospital of Central South University, Changsha 410008, China; ^2^ Binhai Genomics Institute, BGI-Tianjin, BGI-Shenzhen, Tianjin 300308, China; ^3^ Tianjin Translational Genomics Center, BGI-Tianjin, BGI-Shenzhen, Tianjin 300308, China; ^4^ Wuhan National Laboratory for Optoelectronics, Huazhong University of Science and Technology, Wuhan 430074, China; ^5^ BGI-Shenzhen, Shenzhen 518083, China; ^6^ James D. Watson Institute of Genome Sciences, Hangzhou 310058, China

**Keywords:** hypopharyngeal cancer, whole-exome sequencing, mutations, epigenetic, therapeutic target

## Abstract

Hypopharyngeal cancer (HPC) frequently presents at an advanced stage, resulting in poor prognosis. Although combined surgical therapy and chemoradiotherapy have improved the survival for patients with HPC over the past 3 decades, the mortality rate in late-stage diagnosis of HPC is unsatisfactory. In this study, we performed whole-exome sequencing (WES) of 23 hypopharyngeal tumor and paired adjacent normal tissue to identify novel candidate driver genes associated with hypopharyngeal carcinoma. We identified several copy number variants (CNVs) and 15 somatic mutation genes that were associated with hypopharyngeal carcinoma. Mutations in nine new genes (PRB4, NSD1, REC8, ZNF772, ZNF69, EI24, CYFIP2, NEFH, KRTAP4-5) were also indentified. PRB4 and NSD1 expression were significantly upregulated in hypopharyngeal carcinoma, which was confirmed in an independent cohort using IHC. There was a positive relationship between PRB4 and NSD1. Downregulation of PRB4 by siRNA could inhibit cell growth, colony formation and cell invasion. Notably, we here demonstrate that NSD1 could bind to the promoter regions of PRB4 and activate promoter activity by reducing the binding of H3K27me2 and increasing the binding of H3K36me2 on PRB4 promoter. In summary, we pinpoint the predominant mutations in hypopharyngeal carcinoma by WES, highlighting the substantial genetic alterations contributing to hypopharyngeal carcinoma tumorigenesis. We also indentify a novel epigenetically regulatory between PRB4 and NSD1 that contribute to hypopharyngeal carcinoma tumorigenesis. They may become potential prognostic biomarkers and therapeutic target for hypopharyngeal carcinoma treatment.

## INTRODUCTION

Hypopharyngeal cancer (HPC) mainly originated in the pyriform sinus, followed by sites not otherwise specified, and the posterior hypopharyngeal wall [1]. These tumors frequently present at an advanced stage, and display early submucosal spread, resulting in poor prognosis, among the worst of all head and neck subsites [2]. Surgery is difficult to achieve due to the multifocal disease and early lymphatic spread. Although combined surgical therapy and chemoradiotherapy have improved the survival for patients with HPC over the past 3 decades, the mortality rate in late-stage diagnosis of HPC is unsatisfactory [3]. Detection of HPC at an earlier stage would be beneficial to patients. Many molecules, such as p16, bcl-2, microRNAs, and cyclin-D1, have been evaluated as candidate biomarkers for HPC [4, 5], but none has been widely used in practice because of each belongs to signaling pathways of multiple known or unknown proteins [6]. Therefore, more effective biomarkers for early diagnosis of HPC are necessary. And understanding of the molecular mechanisms involved in HPC development, progression, and treatment response is also necessary.

Recently, next generation sequencing has become very useful tools for identifying gene alterations and novel biomarkers and therapeutic targets [7]. For instance, Sawada G et al performed whole-exome sequence analysis of tumor and nontumor esophageal tissues collected from 144 patients with esophageal squamous cell carcinoma, and found that many tumors contained mutations in genes that regulate the cell cycle (TP53, CCND1, CDKN2A), epigenetic processes and receptor-tyrosine kinase-phosphoinositide 3-kinase signaling pathways (PIK3CA, EGFR, ERBB2) [8]. Whole exome sequencing on cisplatin-resistant metastatic squamous cell carcinoma of head and neck tumors revealed that inactivation of REV3L may inform treatment options in patients of recurrent squamous cell carcinoma of head and neck tumors [9]. Thus, whole exome sequencing can provide comprehensive insights into the mutational signatures of cancers and identify markers for early diagnosis and potential therapeutic targets. However, the mutations in hypopharyngeal cancer remain largely unknown.

In this study, we performed whole-exome sequencing (WES) of 23 hypopharyngeal tumor and paired adjacent normal tissue and confirmed the expression of NSD1 and PRB4 in an independent cohort containing 88 hypopharyngeal tumor and 36 adjacent normal tissues. In addition, we further investigate the function of PRB4 in hypopharyngeal tumor and the underlying mechanisms.

## RESULTS

### Clinicopathologic features of the case for whole-exome sequencing

**Figure 1:**
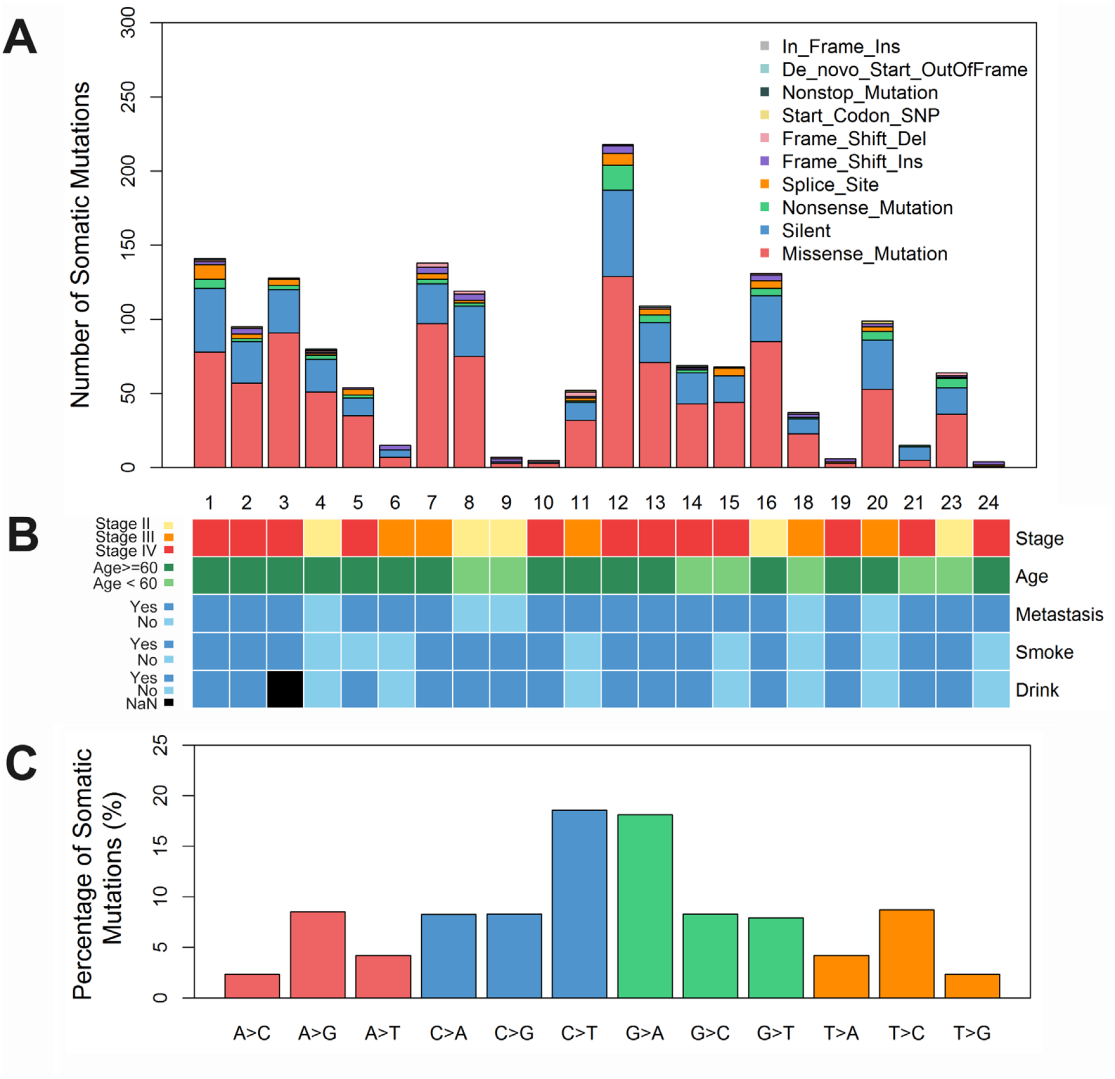
Somatic mutation of hypopharyngeal cancer tissues **(A)** A summary of all kinds of somatic mutations in hypopharyngeal cancer tissues. Each bar represents individual mutations in each tissue. **(B)** The clinicopathological features of hypopharyngeal cancer patients. **(C)** The bars show percentage of somatic single nucleotide variations identified by whole exome sequencing in hypopharyngeal cancer tissues, compared to adjacent tissues. del, deletion; Ins, insert.

Twenty-four pared adjacent and tumor tissues from patients with hypopharyngeal carcinoma were collected to perform whole-exome sequencing. As the low quality of DNA extracted from No. 22 tissue, this tissue was excluded in analysis. The information of patients on demography, risk factors, clinical stage, and histopathological features were recorded. All the patients were male, age was between 41-80 years old, 64% (14/23) were exposed to tobacco and alcohol, 78.2% (18/23) presented at advanced stage III/IV, and nodal metastasis (Figure 1B).

### Whole-exome sequencing

Whole-exome sequencing of 23 hypopharyngeal carcinoma tumors and matched adjacent normal tissues from patients was performed. Approximately 3.32∼4.82 Gb of cleaned sequencing data were obtained for each sample. About 99.02% of the sequencing reads were mapped to the human genome 19, with mean 57× (range 34–80×) sequencing depth. The somatic mutations mainly included missence mutation (50-70%), Silent mutation (20-30%), and nonsence mutation (1-5%) (Figure 1A). The most common type of mutation in the exomic region was C>T and G>A transition, followed by A>G and T>C transition (Figure 1C).

**Figure 2:**
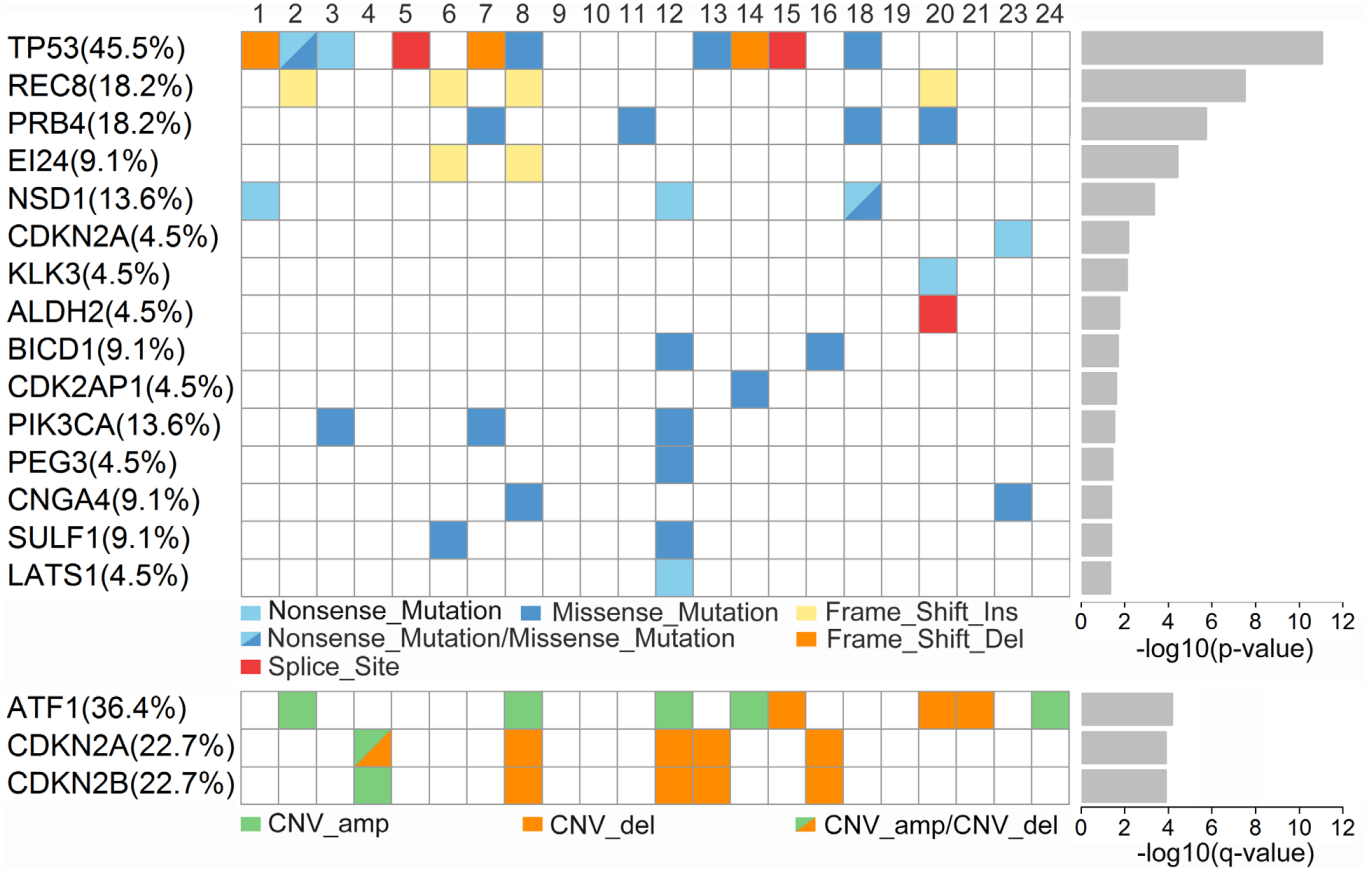
Mutations of genes in hypopharyngeal cancer tissues Upper chart indicates number and type of mutations for each gene. Lower chart shows the copy number variations (CNVs) of ATF1, CDNK2A and CDNK2B. amp, amplification; del, deletion; Ins, insert.

We searched for enrichment of somatically mutated and CNVs (amplication or deletion) genes with p<0.05 in the KEGG pathways including the Cell Cycle signaling, PI3K signaling, RAS signaling, Hippo signaling, and cell adhesion through GSEA algorithm combined with mutational analysis. PI3K signaling pathway was identified as the most significantly altered (FDR≤0.05). Fifteen somatic mutations (TP53, REC8, PRB4, EI24, NSD1, CDKN2A, KLK3, ALDH2, BICD1, CDK2AP1, PIK3CA, PEG3, CNGA4, SULF1, LATS1) and three CNVs genes (ATF1, CDKN2A, CDKN2B) were identified (Figure 2). Hence, these data shed light on the essential role of dysregulation of these critical pathways in tumorigenesis of hypopharyngeal carcinoma.

As shown in Table 1, mutations resulted in down-regulation or up-regulation of genes expression. In order to explore the possible predicting biomarkers of hypopharyngeal carcinoma, we especially concerned with the amplication genes. Among them, the somatic mutation of PRB4 and NSD1 caused gene activation (Fold change > 5). Other activated genes included SYCP1 PDE4DIP, KIAA1383, DUSP19, hsa-mir-28, ATF1, EEA1 and SYCP2. And the top ten of downregulated genes included EPHA5, SERF1B, PCDHB9, CDKN2A, EFNB2, IL9R, hsa-mir-1302-9, LOC100129138, FOXD4L4, STX19 (Table 1).

**Table 1:** The top ten upregulated and downregulated genes in adjacent and tumor tissues

Upregulated genes	cytoband	Expression		Fold change (tumor vs. adjacent)
		adjacent	tumor	
PRB4	12p13.2	376655	2069567	5.52
NSD1	5q35.3	45497	311837	6.91
SYCP1	1p13.2	121440	451095	3.73
PDE4DIP	1q21.1	323511	974559	3.04
KIAA1383	1q42.2	116503	266925	2.30
DUSP19	2q33.1	227069	948717	4.24
hsa-mir-28	3q25.2	252910	729638	2.95
ATF1	12q13.12	193112	586193	3.02
EEA1	12q22	200547	723458	3.64
SYCP2	20q13.33	241605	996799	4.17
Downregulated genes				
EPHA5	4q13.1	592005	199657	0.34
SERF1B	5q13.2	817299	169158	0.21
PCDHB9	5q31.3	958396	296799	0.31
CDKN2A	9p21.3	788582	304950	0.39
EFNB2	13q33.3	996799	391073	0.39
IL9R	Yq11.223	1076166	537999	0.50
hsa-mir-1302-9	9p24.3	816827	149433	0.18
LOC100129138	1p21.1	527704	131291	0.25
FOXD4L4	9p11.2	387398	131880	0.34
STX19	3p12.1	811353	311972	0.38

### Upregulation of PRB4 and NSD1 is predictive of a poor outcome of patient with hypopharyngeal carcinoma

**Figure 3:**
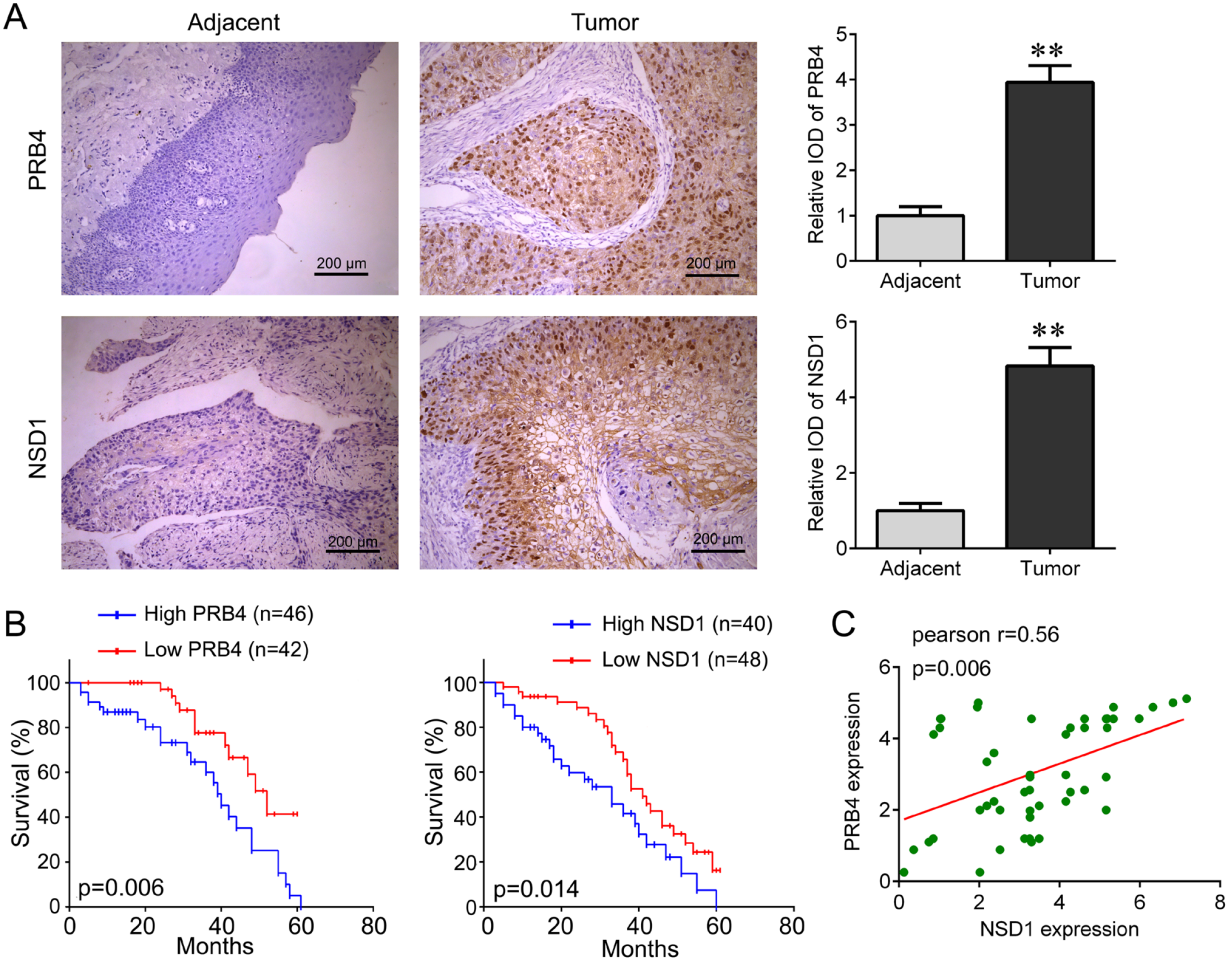
PRB4 and NSD1 are increased in human hypopharyngeal carcinoma tissues **(A)** PRB4 and NSD1 were identified as positively expressed by immunohistochemical staining analysis and their expression was significantly higher in human hypopharyngeal carcinoma tissues (n=88) than in adjacent tissues (n=36). Scale bar, 200 μm. **p<0.01. **(B)** The relationship between PRB4 expression (left), NSD1 expression (right) and overall survival rate of hypopharyngeal carcinoma patients. The patients with high PRB4 or NSD1 expression have a shorter survival time than that with low PRB4 or NSD1 expression. **(C)** The correlation between PRB4 and NSD1 expression in human hypopharyngeal carcinoma tissues.

We focused on the role of PRB4 and NSD1 in hypopharyngeal carcinoma. An independent cohort containing 88 tumor tissues and 36 adjacent tissues was used to confirm their expressions using IHC staining. We found that PRB4 and NSD1 were dramatically increased in hypopharyngeal carcinoma tissues compared with adjacent tissues (Figure 3A). And the Kaplan-Meier survival cure showed that the patients with high PRB4 and NSD1 expression had shorter survival time than those with low PRB4 and NSD1 expression (Figure 3B). We also found that the expression of PRB4 was positively correlated with NSD1 expression (Figure 3C). In addition, PRB4 and NSD1 expression was associated with tumor size (p=0.010), metastasis (p=0.029) and clinical stage (p=0.005), but both their expressions were not associated with smoking and alcohol (Table 2). Moreover, we investigated the factors that could predict the prognosis of hypopharyngeal cancer patients by using Cox proportional hazard regression model for the univariate and multivariate analysis. Univariate analysis data indicated that the PRB4 level (P=0.013) and NSD1 level (P=0.010), as well as the tumor size (P=0.027), metastasis (P=0.006), and clinical stage (P=0.008) was significantly associated with the survival (Table 3). Moreover, as demonstrated in Table 4, the PRB4 level (P=0.027), NSD1 level (P=0.018), tumor size (P=0.030), metastasis (P=0.012) and clinical stage (P=0.021) were found to be independent factors for predicating the prognosis of hypopharyngeal cancer patients. Thus, the results suggest that PRB4 and NSD1 might contribute to the development of hypopharyngeal cancer.

**Table 2:** Clinical association between PRB4 and NSD1 expression and clinicopathological variables in hypopharyngeal cancer patients

Variable	PRB4 expression		p	NSD1 expression		P
	Low (n=42)	High (n=46)		Low (n=48)	High (n=40)	
Age			0.834			0.834
<60	20	23		24	19	
≥60	22	23		24	21	
Gender						
Male	42	46	-	48	40	-
Female	0	0				
Tumor size			0.018			0.010
<3cm	25	15		28	12	
≥3cm	17	31		20	28	
Metastasis			0.005			0.029
No	24	12		25	11	
Yes	18	34		23	29	
Stage			0.030			0.005
I-II	23	14		27	10	
III-IV	19	32		21	30	
Smoking			0.598			0.602
No	10	8		11	7	
Yes	32	38		37	33	
Alcohol			0.659			0.074
No	14	18		13	19	
Yes	28	28		35	21	

Metastasis, lymph node metastasis or distant metastasis; stage, clinical stage; p, chi-square test.

**Table 3:** Univariate analysis of prognostic factors of hypopharygeal cancer

Variable	Hazard ratio	p value
Age (≥60/<60)	1.23	0.671
Tumor size (≥3cm/<3cm)	2.35	0.027
Metastasis (Yes/No)	4.35	0.006
Clinical stage (III-IV/I-II)	4.26	0.008
PRB4 expression (High/Low)	3.02	0.013
NSD1 expression (High/Low)	3.65	0.010

**Table 4:** Multivariate analysis of independent prognostic factors of hypopharygeal cancer

Variable	Hazard ratio	p value
Tumor size	2.53	0.030
Metastasis	3.87	0.012
Clinical stage	3.11	0.021
PRB4 expression	2.63	0.027
NSD1 expression	3.73	0.018

### Knockdown of PRB4 by siRNA inhibits the cell proliferation and invasion of hypopharyngeal cancer cells

**Figure 4:**
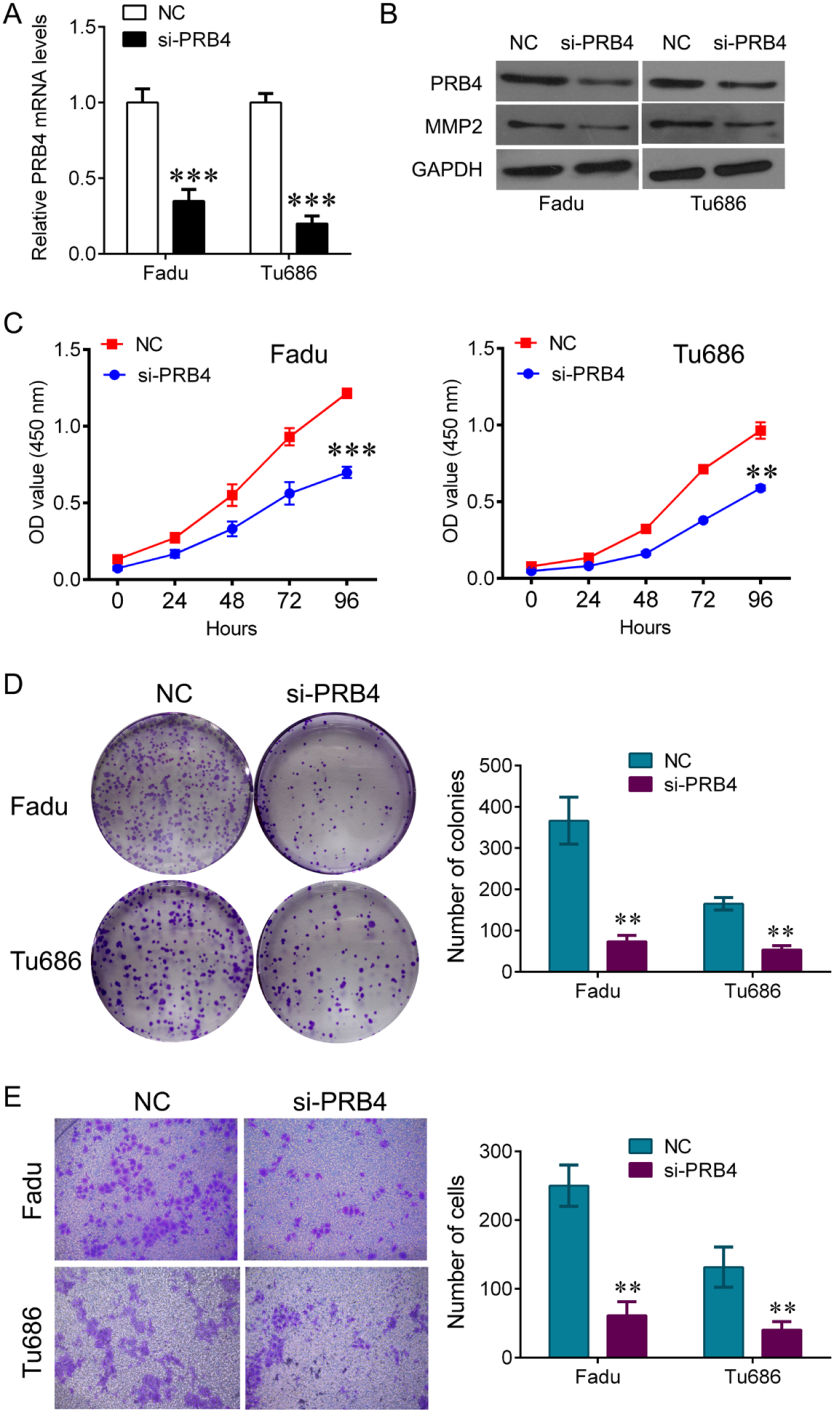
knockdown of PRB4 represses Fadu and Tu686 cells growth and invasion **(A)** QPCR detected the expression of PRB4 in Fadu and Tu686 cells after si-PRB4 transfection. **(B)** Western blot detected the expression of PRB4 and MMP2 in Fadu and Tu686 cells after si-PRB4 transfection. **(C)** CCK-8 was used to measure the cells proliferation in Fadu and Tu686 cells after si-PRB4 transfection. **(D)** Colony formation assay was used to measure the ability of colony formation in Fadu and Tu686 cells after si-PRB4 transfection. **(E)** Transwell assay was used to measure the ability of invasion in Fadu and Tu686 cells after si-PRB4 transfection. Data are expressed as mean ± standard deviation. **P<0.01, ***P<0.001 vs. negative control.

To investigate whether PRB4 affected the growth of Fadu and Tu686 cells, the cells were transfected with PRB4 siRNA. QPCR and western blot were used to confirm knockdown of PRB4 expression (Figure 4A, 4B). As shown in Figure 4C and 4D, PRB4 downregulation significantly impeded the cell viability and the ability of colony formation in Fadu and Tu686 cells. A corresponding effect on cell invasion was also observed in transwell assay, which showed a significant reduction of invasive cells in Fadu and Tu686 cells compared with the respective NC group (Figure 4E), and the expression of MMP2 was also significantly decreased by PRB4 downregulation (Figure 4B).

### NSD1 epigenetically activates PRB4 expression and PI3K signaling

**Figure 5:**
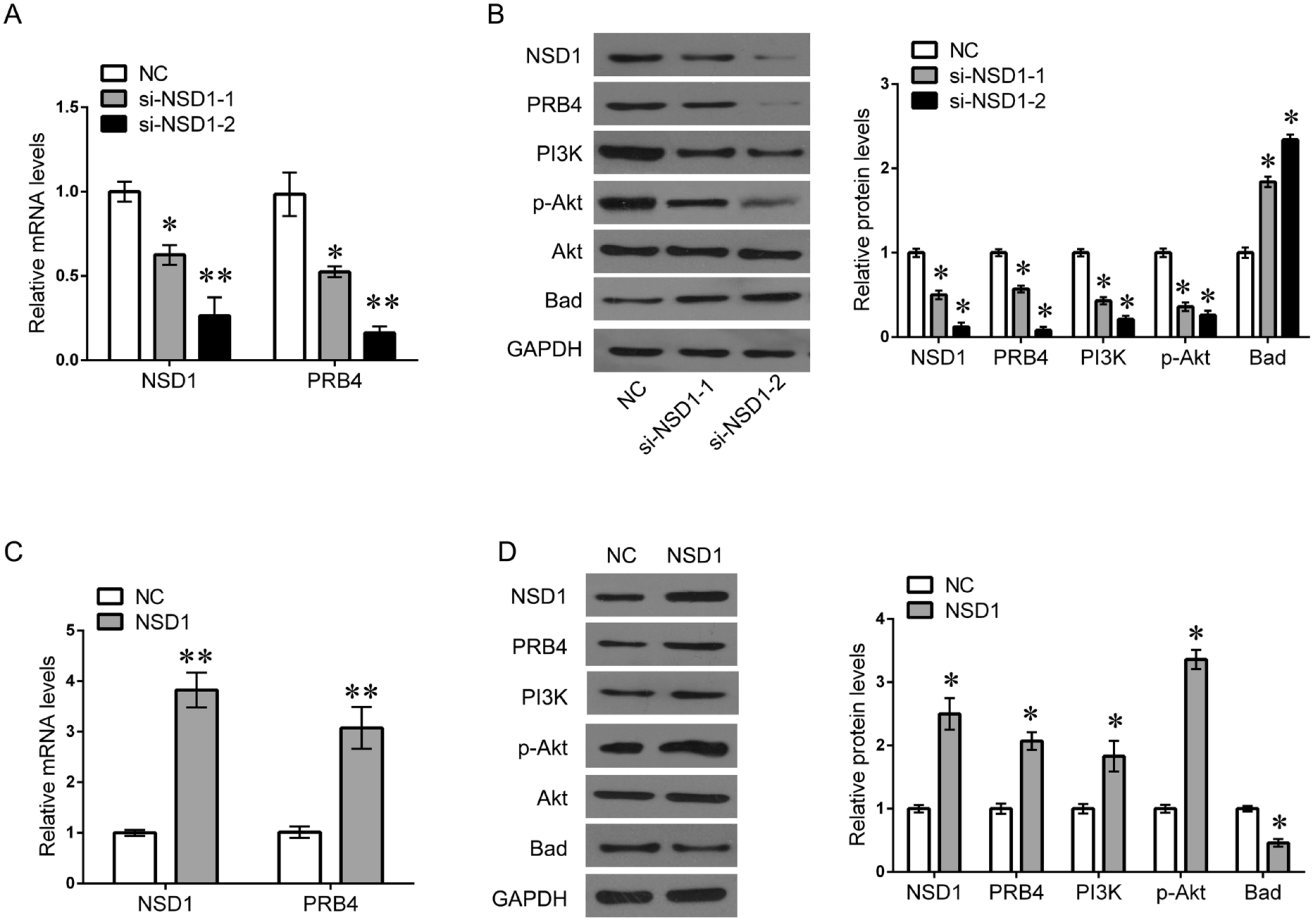
NSD1 activates PRB4 and PI3K signaling **(A)** QPCR detected the expression of PRB4 and NSD1 in Fadu cells after si-NSD1 transfection. **(B)** Fadu cells were transfected with si-NSD1 for 48 h. And then the expression of NSD1, PRB4, PI3K, Akt, p-Akt and Bad protein were measured by western blot, and quantification. **(C)** QPCR detected the expression of PRB4 and NSD1 in Fadu cells after NSD1 transfection. **(D)** Fadu cells were transfected with NSD1 for 48 h. And then the expression of NSD1, PRB4, PI3K, Akt, p-Akt and Bad protein were measured by western blot, and quantification. Data are expressed as mean ± standard deviation. *P<0.05, **P<0.01 vs. negative control.

**Figure 6:**
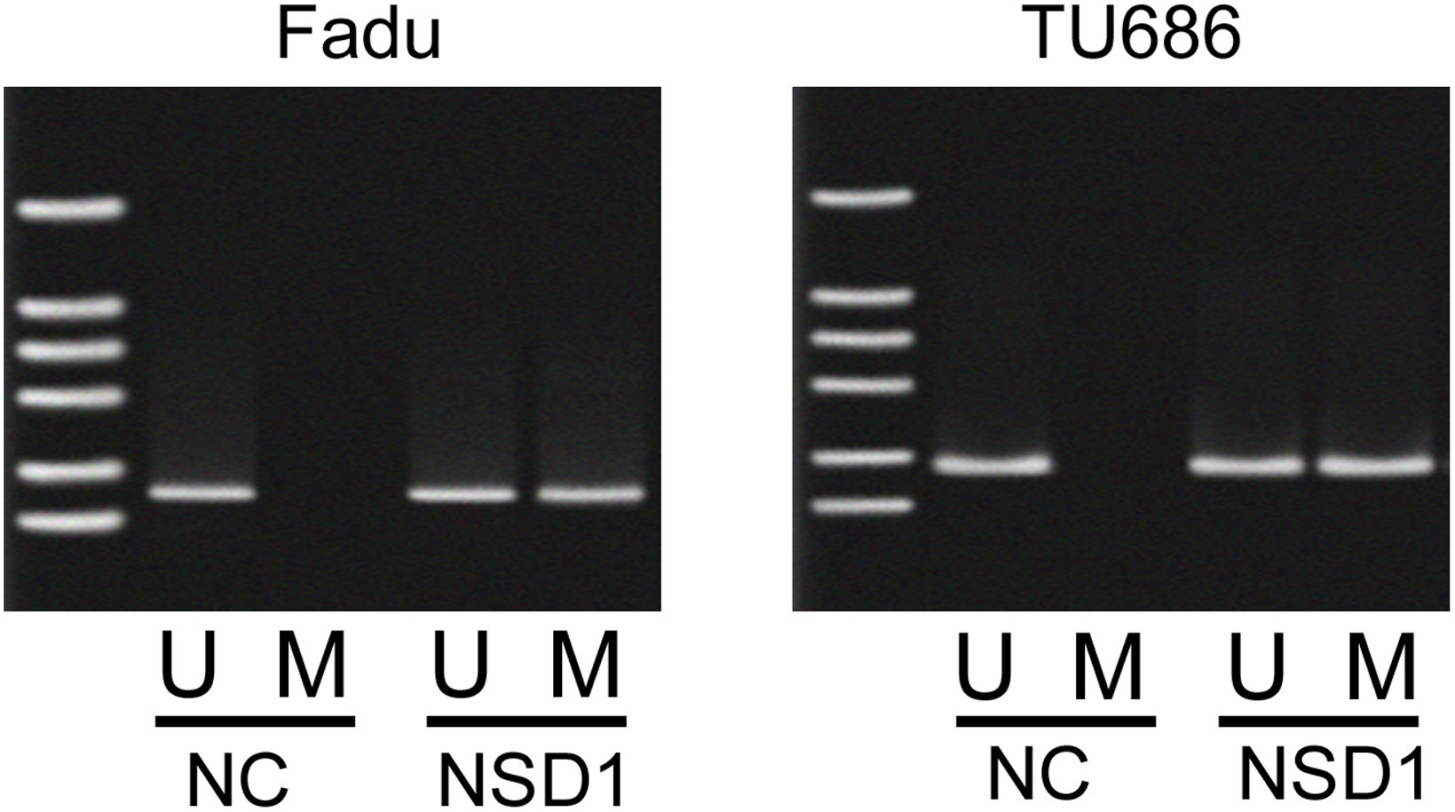
The methylation levels of promoter of PRB4 in Fadu and Tu686 cells MSP assay was performed to determine the methylation levels of promoter of PRB4 after overexpressing NSD1. U, unmethylation; M, methylation.

**Figure 7:**
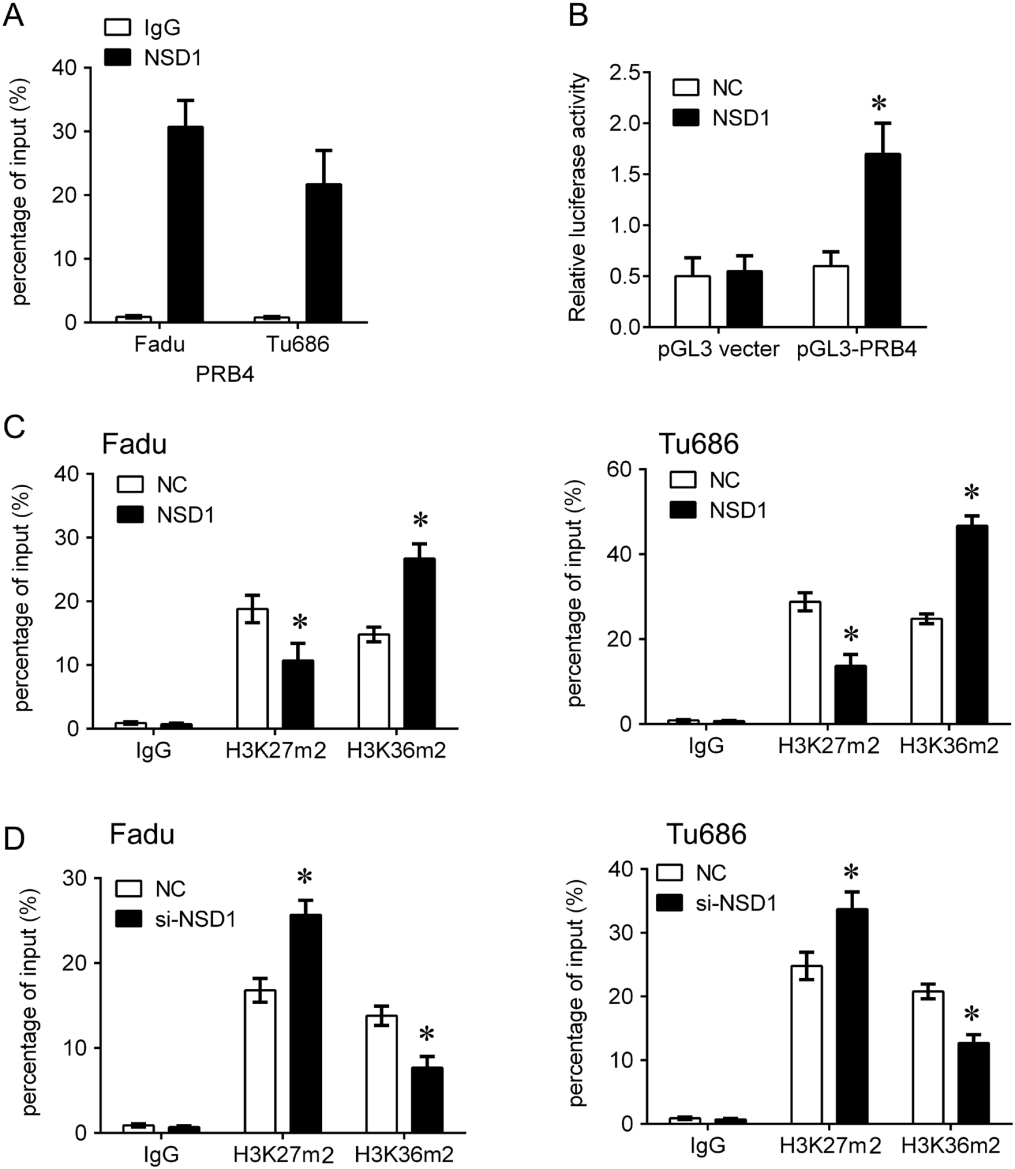
NSD1 epigenetically activates PRB4 by recruiting H3K36m2 **(A)** ChIP-qPCR analysis of NSD1 occupancy in the PRB4 promoter regions in the Fadu and Tu686 cells. IgG was used as a negative control. **(B)** Luciferase reporter analysis of luciferase activity in the Fadu cells cotransfected with pGL3-PRB4 and NSD1 lentivirus or an empty lentivirus. **(C, D)** ChIP-qPCR analysis of H3K27m2 and H3K36m2 occupancy in the PRB4 promoters in the Fadu and Tu686 cells after transfection with NSD1 lentivirus (C) or si-NSD1 (D). IgG was used as a negative control. *P < 0.05 vs. negative control.

Due to the positive correlation between NSD1 and PRB4, we further investigated whether NSD1, a histone transmethylase, could regulate the expression of PRB4. We found that knockdown of NSD1 could significantly decrease the mRNA and protein levels of PRB4, whereas overexpression of NSD1 increased the expression of PRB4 (Figure 5). Interestingly, knockdown of NSD1 significantly inactivated PI3K signaling evaluated by decreased PI3K and phosphorylated Akt expression and increased Bad expression. Overexpression of NSD1 exhibited the opposite effects on PI3K signaling (Figure 5). We further investigated the mechanism by which NSD1 regulates PRB4. We performed MSP to examine whether NSD1 could regulate the methylation status of PRB4 promoter. And we found that NSD1 overexpression hypermethylated PRB4 promoter in Fadu and Tu686 cells (Figure 6). ChIP assay was performed to evaluate whether NSD1 could bind to PRB4 promoter. The results showed that NSD could bind to the promoter regions of PRB4 (Figure 7A). In addition, the promoter region (2000 bp) of PRB4 was inserted into a PGL3 luciferase reporter vector, and dual-Luciferase reporter analysis showed that NSD1 could bind to this region and activate luciferase (Figure 7B). Moreover, we also found that overexpression of NSD1 could reduce the binding of H3K27me2 on PRB4 promoter, while increase the binding of H3K36me2 on PRB4 promoter (Figure 7C). However, knockdown of NSD1 reversed these bindings in Fadu and Tu686 cells (Figure 7D). These results indicate that PRB4 upregulation in hypopharyngeal cancer may be epigenetically activated by NSD1.

## DISCUSSION

In order to better understand the mutations in hypopharyngeal carcinoma, WES was performed in 23 paired tumor and adjacent normal tissues. Our study identifies novel candidate driver genes associated with hypopharyngeal carcinoma. These genes are different from earlier exome-sequencing studies on head and neck squamous cell carcinoma with lower mutation frequency and higher C>T transition predominantly occurred at CpG sites [7, 12], while are similar with esophageal squamous cell carcinoma, which may be due to the same histological origin in esophagus and hypopharyngeal carcinoma and share the same risk factors (alcohol and gastroesophageal reflux).

Moreover, we identified several copy number variants (CNVs) genes were associated with hypopharyngeal carcinoma, one with amplification (ATF1) and two with deletions (CDKN2A, CDKN2B). CDKN2A and CDKN2B encode protein p16^INK4a^ and p15^INK4b^ respectively, which belong to a family of cyclin-dependent kinases (CDKs) inhibitor proteins that can inactivate CDKs [13]. Both of proteins can specially bind to CDK4/CDK6 and subsequently induce an allosteric conformational change to inhibit the formation of the CDK4/6 complex and cyclin D, leading to G1 phase cell cycle arrest [14]. They therefore potentially act as tumor suppressors, and their inactivation corresponds to human carcinogenesis. The loss of CDKN2A and CDKN2B was observed in various cancers through deletion, inactivating mutations, epigenetic silencing or post-translational modification [15, 16]. In this study, CDKN2A was found down-regulated expression (61.0%) and point mutation (4.5%), suggesting CDKN2A and CDKN2B deficiency and inactivation may contribute to hypopharyngeal carcinoma progression.

In addition, we identified 15 SMGs associated with hypopharyngeal carcinoma. Among these genes, TP53 and PIK3CA are well-known cancer-associated genes. TP53 is known as the ‘‘gatekeeper’’ gene in the cellular defense against genotoxic damage and functions as a critical tumor suppressor. We found that nearly half of hypopharyngeal carcinoma (45.5%) has TP53 mutations, supporting that the loss of function of TP53 is a key initiating or tumor promoting event in hypopharyngeal carcinoma. PIK3CA gene, an important molecule in the PI3K (RAS/PI3K/AKT) pathway, is the main genetic driving force of this pathway in human cancers. Mutations of PIK3CA were found in 13.6% of hypopharyngeal carcinoma, and the E545K and H1047R mutant sites, two of hotspots mutations of PIK3CA, have been demonstrated that they can increase PIK3CA kinase activity and promote cellular transformation [17, 18]. These findings indicate aberrantly PIK3CA activation is another fundamental mechanism in hypopharynx tumorigenesis.

We also discovered nine new genes (PRB4, NSD1, REC8, ZNF772, ZNF69, EI24, CYFIP2, NEFH, KRTAP4-5) associated with hypopharyngeal carcinoma. Proline-rich proteins are the most conserved oral salivary proteins among mammals. The high polymorphism of proline-rich proteins gives an important contribution to the high heterogeneity, possibly involved in different biological activities [19]. Mutations of proline rich protein BstNI subfamily (PRB) 4 and 1 were found in primary gastric cancer and matched peritoneal metastatic cancer tissues [20]. However, the functions of PRB4 and NSD1 (nuclear receptor binding SET domain protein 1) are not clear in hypopharyngeal carcinoma. Their expression was significantly upregulated in hypopharyngeal carcinoma estimated by WES, which was confirmed in an independent cohort using IHC. There was a positive relationship between PRB4 and NSD1. We knocked down PRB4 expression hypopharyngeal carcinoma cells, and found that downregulation of PRB4 could inhibit cell growth, colony formation and cell invasion, and we speculate that the mutations in this gene enhanced it oncogenic effects. NSD1 is a SET-domain histone methyltransferase that methylates lysine 36 of histone 3 (H3K36) [21], which is associated with chromosomal instability by methylation [22]. Initially, NSD1 mutations were found to lead to the Sotos syndrome. More recently, many studies show that the NSD1 were overexpressed, amplified or somatically mutated in multiple types of cancer, suggesting their critical role in cancer [23, 24]. Its dysfunction results in epigenetic aberrations which play a fundamental role in oncogenesis [25]. Notably, we here demonstrate that NSD1 positively regulates PRB4 expression and PI3K signaling. Further mechanism investigation reveals that NSD could bind to the promoter regions of PRB4 and activate promoter activity by reducing the binding of H3K27me2 and increasing the binding of H3K36me2 on PRB4 promoter. These results indicate that PRB4 upregulation in hypopharyngeal cancer may be epigenetically activated by NSD1. Therefore, PRB4 and NSD1 mutations might contribute to hypopharyngeal carcinoma tumorigenesis.

In summary, we pinpoint the predominant underlying mutational processes in hypopharyngeal carcinoma by WES, highlighting the substantial genetic alterations contributing to hypopharyngeal carcinoma tumorigenesis. We also indentify a novel epigenetically regulatory between PRB4 and NSD1 that contribute to hypopharyngeal carcinoma tumorigenesis. They may become potential prognostic biomarkers and therapeutic target for hypopharyngeal carcinoma treatment.

## MATERIALS AND METHODS

### Samples and clinical data

This study was approved by the Xiangya Ethics Committee of the Central South University. Written informed consent was obtained from individual patients who participated in the study. All human frozen and paraffin-embedded tissues as well as clinical information were obtained from The Xiangya Hospital of Central South University. Twenty-four tumor tissues and matched adjacent tissues obtained from patients with hypopharyngeal carcinoma from January 2011 to December 2015 were used for whole exome sequencing (WES) analysis. After resection, samples were immediately preserved in liquid nitrogen. The tissues were verified using routine H&E staining by pathologists. The cancer tissues containing more than 90% squamous cells and their paired adjacent tissues containing more than 95% normal cells were included for whole-exome sequencing. One pair of samples was excluded due to low quality, and 23 pairs of tumor-adjacent frozen biopsies were applied for WES. Another independent cohort containing 88 tumor tissues and 36 normal adjacent tissues was used for verification of candidate genes by immunohistochemistry (IHC) analysis.

### Whole-exome sequencing

Genomic DNA samples were extracted from tumor and adjacent tissues obtained from patients with hypopharyngeal carcinoma using TIANamp Genomic DNA Kits (Qiagen, Valencia, CA, USA). Qubit^®^ 2.0 Fluorometer (Life technologies) was used to evaluate DNA quality and quantity. Two micrograms of genomic DNA from each sample was used for generating the sequencing library using the Agilent SureSelect Library Prep Kit according to the manufacturer’s protocol. Exome regions were captured and enriched with the SeqCap EZ Human Exome Library v3.0 (Nimblegen). Captured DNAs were subjected to high-throughput sequencing using an Illumina HiSeq 2000/2500 platform to generate 101 bp paired-end reads.

### Variant calling and prioritization

Base calling was performed using the Real-Time Analysis software (Illumina). The whole-exome sequencing data were analyzed with the following criterion of quality control:1) reads contaminated by adapter sequences together with their mate pair reads were removed; 2) reads with more than 50% of low-quality bases(Q<5) were removed; and 3) reads with a high N rate (>0.1) and their mate pair reads were removed. BWA (Burrows-Wheeler Aligner) was used to align clean reads against to the human reference assembly hg19. SAM tools were used for conversion of SAM files to BAM files. Reads with multiple mapping loci in the genome and reads with more than 3 mismatches, with more than 1 gap, or with a gap more than 20 bases long were removed. More than one file from a patient was merged for further genomic analysis. The duplicated reads derived from PCR amplification were marked using Picard tools (http://broadinstitute.github.io/picard/). Local realignments and base quality recalibrations were performed using Genome Analysis Toolkit (GATK).

### Statistical inferences on nature of genomic alteration

The Mutect algorithm and VarScan algorithm were applied to identify somatic single-nucleotide variants (SNV) in targeted regions. All these SNVs were annotated with Oncotator (http://www.broadinstitute.org/cancer/cga/oncotator/). SNVs were filtered according the following conditions:

1) SNV was detected by more than one software; 2) Filter SNV: alternative allele depth >=5 and total depth >= 30 in cancer and total depth >= 30 in normal, 1000 genome was freq<=0.05; AndVarScan was used to identify somatic Indels with the default parameters and subjected to filter with the following criterion of somatic indels: Varscan Somatic and Total Detph>= 50 in Normal and Tumor Sample.

### Copy number variation

To identify CNAs (copy number alterations), VarScan was used to compare read depths between tumors and matched adjacent samples for contiguous regions of coverage. And then a circular binary segmentation (CBS) algorithm was used to delineate segments using DNA copy package, pick out significant change-points and merge these regions with mergeSegment.pl.

### Immunohistochemistry (IHC) and staining evaluation

IHC staining was performed as previously described [10, 11]. Tissues sections were deparaffinized in xylene and rehydrated in alcohol, and then pretreated with citrate buffer (10 mmol/l, pH 6.0) for 20 min at 100°C in a microwave oven; 3% hydrogen peroxide was used to block endogenous peroxidase activity for 15 min at room temperature. After that, nonspecific binding sites were blocked by 10% normal goat serum for 30 min at 37°C. The sections were incubated with antibody (rabbit polyclonal anti-PRB4, 1:200 dilution, rabbit monoclonal anti-NSD1 1:200 dilution, Abcam) overnight at 4°C. The sections incubated with PBS instead of the primary antibody were used as negative controls. Sections were incubated with biotinylated goat anti-rabbit IgG (1:1000 dilution, Zhongshan Chemical) for 20 min at 37°C after rinsing with PBS. Finally, tissue sections were incubated with 3', 3'-diami-nobenzidine (DAB; Maixin, Fuzhou), then counterstained with Harris modified hematoxylin (Zhongshan Chemical). For evaluating the expression of PRB4 and NSD1 in tissues, the integral optical density (IOD) was obtained by ImageJ (National Institutes of Health, USA). To compare the expression of PRB4 and NSD1 between adjacent and tumor tissues, the IOD of PRB4 and NSD1 expression was normalized to the average score of them in normal tissues. The IOD of PRB4 and NSD1 higher than their mean IOD in adjacent tissues was identified as high expression, otherwise as low expression.

### Cell lines

Two kinds of hypopharyngeal carcinoma cell lines, Fadu and Tu686 cells, were obtained from Cell Bank of Chinese Academy of Sciences (Shanghai, China). All cells were grown in DMEM/F-12 (Gibco, Grand Island, NY, USA) supplemented with 10% fetal bovine serum (Gibco) at 37°C in 5% CO_2_. Knockdown of PRB4 and NSD1 in Fadu and Tu686 cells was achieved by transfection with lentivirus containing PRB4 siRNA or NSD1 siRNA (si-PRB4, si-NSD1-1, siNSD1-2, Genepharma, Shanghai, China) using Lipofectamine 2000 (Invitrogen, CA, USA). Overexpression of NSD1 was achieved by using lentivirus containing NSD1 expressed plasmid (GeneCopoeia, Guangzhou, China) using Lipofectamine 2000 (Invitrogen, CA, USA). The cells transfected with empty lentivirus were used as negative control. Cells were plated in 6-well clusters or 96-well plates and transfected for 24 h or 48 h. Transfected cells were used in further assays or protein extraction.

### RNA extraction and SYBR green quantitative PCR analysis

Total RNA was extracted from cells using Trizol reagent (Invitrogen, CA, USA). The expression of PRB4 and NSD1 was measured by SYBR green qPCR assay (Takara, Dalian, China) according to manufacturers’ instructions. Expression of β-actin was used as an endogenous control. The primers were used as followings: PRB4, sense, CCAGTCCCAAGGAAAGCCAC, anti-sense, CCTTGTTCCAATG TCACGGC; NSD1, sense, GGATGGATCAGACCTGTGAACT, anti-sense, TCTGG ATCATCCGAAAGGGCTG; β-actin, sense, AGGGGCCGGACTCGTCATACT, anti-sense, GGCGGCACCACCATGTACCCT. QPCR was performed at the condition: 95.0 °C for 3 min, and 39 circles of 95.0 °C for 10s and 60 °C for 30 s. Data were processed using 2-ΔΔCT method.

### Western blotting

Cultured or transfected cells were lysed in RIPA buffer with 1% PMSF. Western blot was performed on 10% SDS-PAGE using Mini-PROTEAN^®^ Tetra Cell Systems (Bio-Rad). Proteins were transferred onto polyvinylidine difluoride (PVDF) membranes (Immobilon, Millipore). Membranes were incubated with anti-PRB4 rabbit polyclonal antibody (Abcam), anti-NSD1 rabbit monoclonal antibody (Abcam) or PI3K rabbit monoclonal antibody (Cell Signaling) or Akt mouse monoclonal antibody (Cell Signaling) or phospho-Akt rabbit monoclonal antibody (Cell Signaling) or Bad Rabbit monoclonal antibody (Cell Signaling) at 1:1000 dilution, or GAPDH specific antibody (Sigma-Aldrich) at 1:5000 dilution at 4°C overnight. Signals were visualized using ECL Substrates (Millipore, MA, USA).

### Cell viability assay

Cell viability was detected using 2-(2-methoxy-4-nitrophenyl)-3-(4-nitrophenyl)-5- (2,4-disulfophenyl)-2H-tetrazolium, monosodium salt (CCK-8 Kit; Beyotime biotech Co. Ltd., Hangzhou, China). CCK-8 solution (10 μl) was added to each well of 96-well plates with the same amount of culture fluid. CCK-8 solution without cells was used as blank control. Optical density was determined at the time points on a microtiter plate reader at 450 nm.

### Invasion assay

After indicate treatments, equal cell numbers of the Fadu (1×10^6^ cells/chamber) or Tu686 (0.5×10^6^ cells/chamber) cell lines for each condition were placed in deprivation media in the upper chamber (8.0 μ m pores, Corning, Inc) coated with Matrigel (BD Biosciences). Deprivation media was placed in the lower chamber. Cells were allowed to invade for 48 h. The cells remaining in the upper chamber were removed from the upper chamber by a cotton swab. After fixation with 4 % formalin and staining with Differential Quik Stain (Polysciences, Inc), invaded cells were acquired on an inverted Olympus IX81 microscope (Olympus, Tokyo, Japan). To evaluate the invasive ability, the invaded cells were counted.

### Colony formation assay

Following a 48 hour treatment with PRB4 siRNA, cells were seeded at 300 cells per well in 6-well plates containing complete DMEM/F-12 and incubated at 37°C and 5% CO _2_ for 15 days. On day 15, cells were fixed with 4% polyformaldehyde for 15 min and stained with 1% crystal violet. The experiments were performed in triplicate, and the numbers of colonies containing more than 50 cells were counted.

### Measurement of PRB4 promoter methylation status by methylation specific PCR (MSP)

Genomic DNA was extracted using the Qiagen FFPE DNA Kit (Qiagen, CA, USA). Genomic DNA (1 μg per sample) was modified with bisulfite using the EZ DNA Methylation-Gold Kit (Zymo, Orange County, CA, USA) according to the manufacturer's instructions. Methylation-specific PCR (MSP) was performed on bisulfate-treated DNA. The primers used were un-methylated PRB4 forward, TAATATGATATTGTATGGTTTTTGT, and reverse, AACTACCCCACAACCTACTCAAA; and methylated PRB4 forward: TGGTAATATGATATCGTATGGTTTTC, and reverse, AAACTACCCCACAACCTACTCG. The annealing temperature was 67.5 °C for methylated-PCR and 65°C for un-methylated-PCR, with 35 cycles used for each.

### Luciferase reporter assay

The PRB4 promoter region (2000 bp) was synthesized and inserted into a pGL3-basic vector (Promega, Madison, WI, USA). The successful constructs were verified by DNA sequencing. The Dual-Luciferase Assay Kit was used to assess luciferase activities, following manufacturer’s protocol. The cells were plated in 96-well clusters, then cotransfected with 100 ng pGL3-basic vector or pGL3-PRB4, together with NSD1 or negative control. At 48 h after transfection, luciferase activity was detected using a dual-luciferase reporter assay system (Promega, Madison, WI) and normalized to Renilla activity.

### RNA immunoprecipitation (RIP)

RIP assay was used to determine whether PRB4 interacts with or binds to RNA-binding proteins NSD1 in the human hypopharyngeal carcinoma cells. The EZMagna RIP kit (Millipore, Billerica, MA, USA) was used to conduct the RIP experiment, following manufacturer’s protocol. The Fadu and Tu686 cells were lysed using complete RIP lysis buffer; then, the extract was incubated with magnetic beads conjugated with NSD1antibodies or control IgG (Millipore) for 8 h at 4 °C. Next, the beads were washed with washing buffer and incubated with proteinase K at 55 °C for 30 min to remove the proteins. Finally, purified RNA was reverse-transcribed into cDNA and subjected to qPCR analysis to determine the presence of PRB4 using specific primers.

### Chromatin immunoprecipitation (ChIP) assay

The EZ-Magna ChIP kit (EMD Millipore) was used to conduct the ChIP assays in accordance with manufacturer’s protocol, the Fadu and Tu686 cells were fixed with 4% paraformaldehyde and incubated with glycine for 10 min to generate DNA–protein cross-links. Then, the cells were lysed with Cell Lysis Buffer and Nuclear Lysis Buffer and sonicated to generate chromatin fragments. Next, the lysates were immunoprecipitated with Magnetic Protein A Beads conjugated with H3K27me2 (Millipore), or H3K36me2-specific antibodies (Millipore), or IgG as a control. Finally, the precipitated DNA was analyzed by qRT-PCR.

### Statistical analysis

The SPSS Statistics 17.0 package was used to analyze data. All data from 3 independent experiments were expressed as mean ± SD. Chi-square test was used to analyze the association between the level of PRB4 and NSD1 and clinicopathological parameters of hypopharyngeal cancer. Student’s t test was used for statistical analysis in two groups, and data from more than two groups were analyzed by one-way ANOVA. Overall survival (OS) rate was determined by the Kaplan-Meier curve with log-rank test. Cox proportional hazard regression model was used to estimate the independent predicators for the prognosis of hypopharyngeal cancer patients. p< 0.05 was statistically significant.
